# Research on dietary sodium with invalid methods does not advance scientific understanding

**DOI:** 10.1186/s12986-025-01057-1

**Published:** 2026-02-05

**Authors:** Norm R. C. Campbell, Francesco P. Cappuccio

**Affiliations:** 1 Present Address: Department of Medicine, Physiology and Pharmacology and Community Health Sciences, Libin Cardiovascular Institute of Alberta, University of Calgary, Calgary, AB Canada; 2 Warwick Medical School, University of Warwick, Warwick Applied Health, Coventry, UK; 3 Department of Medicine, Foothills Medical Centre – North Tower, 9th Floor, 1403–29th Street NW, Calgary, T2N 2T9 AB Canada

**Keywords:** Dietary sodium, Salt, Hypertension, Blood pressure, Cardiovascular disease, Biomarkers, Methodologic flaws

## Abstract

Wuopio et al. (22:104, 2025) examined associations between urinary sodium excretion and metabolic markers using an invalid method (a spot urine sample and the Kawasaki estimating equation (‘spot method’)). The spot method has both high random and systematic error, and has been shown to produce spurious health outcome and disease association. Due to false controversy generated by studies using spot method, it has been recommended not to be used in research associating dietary sodium to health outcomes by major international and national scientific organizations and experts. Wuopio et al. are aware of the methods limitations and concerns about its use but in our opinion did not address these issues in their manuscript. The spot urine method represents a good example of how an inappropriate research method can undermine scientific understanding and generate false controversy.

Wuopio et al. examined associations between estimated 24-hour urinary sodium excretion and various metabolic markers, using a spot urine sample and the Kawasaki estimating equation (“spot method”) [1]. However, the study was cross-sectional, the timing of urine sampling was not reported, and it was not stated whether the analysis was retrospective [1].

The authors claim that the Kawasaki formula and spot urine method have been validated [1]. However, as they are aware from prior correspondence and a substantial history of critique, this method is not valid for assessing individual sodium intake and is strongly discouraged for use in relation to health outcomes [2–11]. A large body of consistent evidence demonstrates that the spot method introduces both substantial random and systematic error [2–11].

Systematic error has been shown to distort linear associations between dietary sodium and health outcomes (including blood pressure and cardiovascular disease), and can even generate apparent associations when a constant sodium value is substituted into the equation in place of actual values [8–11]. These spurious associations are mathematically predictable, as the Kawasaki equation incorporates urine creatinine, age, sex, height, and weight—factors that themselves are strong predictors of health outcomes. There is no reason to believe that the distortion of associations would not extend to metabolic markers.

Random error is reflected in poor accuracy and reproducibility. For example, in a reanalysis of the PURE study’s Chinese validation cohort, individuals initially classified in the lowest quartile of sodium intake using the spot urine method had a 58% error rate compared with concurrent 24 h urine sodium values, a 104% error rate on a second reassessment, and 68% were reclassified into higher quartiles on second assessment [4].

In the UK biobank study, use of the spot urine method with the Kawasaki equation to assess dietary sodium concluded that “the extreme within-person variability in urinary sodium precluded detection of associations with future blood pressure at resurvey or risk of cardiovascular disease” [11].

Wuopio et al. mischaracterize the lack of validation as merely a “difference of opinion” and inappropriately suggest the method only has the *potential* for bias [1, 12]. However, more than 70 major scientific organizations—supported by numerous internationally recognized methodological experts—have explicitly concluded that the spot method is invalid stating

“The Kawasaki, and other formulae, used to estimate 24 h sodium intake based on spot urine measurements have been shown to result in biased estimates of sodium intake compared with estimates based on 24 hr. urinary collections, to result in a spurious J-shaped association between sodium intake and mortality, and to provide an inaccurate representation of the association between dietary sodium and BP” [13].

Twenty-two of the scientific organizations have also recommended that research using the spot method should not be funded, conducted, or published [3].

Wuopio et al. cite the “PURE” validation study, conducted by one of their co-authors, as support for their approach [1, 14]. However, that study has been subject to extensive critique due to multiple scientific irregularities [4, 6, 15, 16], including:

- Undisclosed alteration of the formula used to assess completeness of 24-hour urine collections, resulting in incomplete collections being misclassified as complete;
- Compromising the reference standard (24 h urine sodium) by including incomplete 24-hour urine samples;
- Excluding 50% of collections deemed incomplete;
- Misrepresenting their method as the Kawasaki approach, by substituting first morning voids for the Kawasaki method that used second morning fasting voids;
- Four published errata in the Kawasaki equation (described in correspondence as typographical errors);
- Selective and incomplete validation analyses;
- Refusal to provide an independent correlation coefficient when requested;
- Relying on a dependent correlation coefficient as evidence of validity, despite the spot sample being a substantive component of the often incomplete 24 h urine sample and Bland–Altman plots showing high systematic error and lack of validity;
- Refusal to release underlying data for independent scrutiny; and.
- Inconsistent disclosure of financial conflicts of interest by several co-authors across publications.

Data from the PURE Chinese validation study are now publicly available. Independent reanalysis has confirmed both high systematic and random error in the method and has revealed multiple analytic errors in the application of the Kawasaki equation—errors not attributable to typographical mistakes [4]. Furthermore, the original Kawasaki validation study, also cited in support of this method, did not include Bland–Altman plots to assess individual-level validity, was based on a small Japanese cohort, and reported correlation values that have not been replicated in most subsequent validation studies [17].

Although convenient, research using the spot method for health outcomes produces false and misleading findings. Ultimately, the greatest “contribution” of the spot method may be its illustration of how low-quality methods can undermine scientific understanding, generate false controversy, and erode public trust in research and public health.

## Data Availability

Not applicable.
